# The cancer multi-disciplinary team from the co-ordinators’ perspective: results from a national survey in the UK

**DOI:** 10.1186/1472-6963-12-457

**Published:** 2012-12-13

**Authors:** Rozh Jalil, Benjamin Lamb, Stephanie Russ, Nick Sevdalis, James SA Green

**Affiliations:** 1 Imperial College, London, W2 1PG, UK; 2 Whipps Cross University Hospital, London E11 1NR, UK; 3 Lister Hospital, Stevenage, Herts, UK; 4 Department of Surgery and Cancer, Imperial College London, 5th Floor Medical School Building, St. Mary’s Hospital, London W2 1PG, UK

**Keywords:** Multidisciplinary team, Cancer, Coordinator, Training, Decision

## Abstract

**Background:**

The MDT-Coordinators’ role is relatively new, and as such it is evolving. What is apparent is that the coordinator’s work is pivotal to the effectiveness and efficiency of an MDT. This study aimed to assess the views and needs of MDT-coordinators.

**Methods:**

Views of MDT-coordinators were evaluated through an online survey that covered their current practice and role, MDT chairing, opinions on how to improve MDT meetings, and coordinators’ educational/training needs.

**Results:**

265 coordinators responded to the survey. More than one third of the respondents felt that the job plan does not reflect their actual duties. It was reported that medical members of the MDT always contribute to case discussions. 66.9% of the respondents reported that the MDTs are chaired by Surgeons. The majority reported having training on data management and IT skills but more than 50% reported that they felt further training is needed in areas of Oncology, Anatomy and physiology, audit and research, peer-review, and leadership skills.

**Conclusions:**

MDT-Coordinators’ role is central to the care of cancer patients. The study reveals areas of training requirements that remain unmet. Improving the resources and training available to MDT-coordinators can give them an opportunity to develop the required additional skills and contribute to improved MDT performance and ultimately cancer care. Finally, this study looks forward to the impact of the recent launch of a new e-learning training programme for MDT coordinators and discusses implications for future research.

## Background

The cancer multidisciplinary team (MDT) is defined as a “group of people of different healthcare disciplines, which meets together at a given time (whether physically in one place, or by video or teleconferencing) to discuss a given patient, and who are each able to contribute independently to the diagnostic and treatment decisions about the patient” [1]. These teams consist of surgeons, radiologists, histopathologists, oncologists, clinical nurse specialists, allied health professionals, and multidisciplinary team coordinators [2].

It has become the standard practise in the UK that treatment plans for cancer patients are made via a MDT meeting and it is now mandatory to treat patients with cancer by specialist MDTs [1]. MDTs are becoming the model of care for patients with cancer worldwide. Research into the specific effects of MDTs on patient outcomes has led to inconclusive results, since other factors including novel treatments, technology and service changes have evolved in parallel [3]. However, MDTs are widely felt to improve communication, coordination and decision-making between healthcare professionals when weighing up treatment options with cancer patients [4].

MDT coordinators are core members of the MDT meeting [5]. Soon after establishing the concept of MDT meetings, a need for a MDT coordinator was visible and a MDT clerk deemed essential to maintain a smooth running and coordinated meeting [6]. The importance of the role of the MDT coordinator has been acknowledged [5-8]. Their duties involve identifying patients for discussion prior to the MDT meeting, organising meetings, facilitating and coordinating the logistics for the MDT meeting. They ensure that an appropriate number of patients are discussed at the meeting. MDT coordinators help in the introduction and changes to the pro-formas used to ensure all patients are discussed, treated appropriately and outcomes are recorded and reviewed. They play a crucial role in bridging the communication gap between the service provider and the patients to enhance the patient-centred care [9]. The role of the MDT Coordinator is relatively new, and as such it is evolving. The pivotal contribution of the MDT coordinator to the effectiveness of an MDT has been acknowledged in a survey of 2000 MDT members in the UK [5].

Research so far has demonstrated that clinical decision-making by cancer MDTs is influenced by many factors including the attendance of team members, the process of case discussion, the information available when making decisions, team leadership, preparation for meetings, facilities and equipment, and the administrative process of auctioning outcomes from meetings [10]. Many, if not all of these factors come into the work of a MDT coordinator. It is, therefore, pivotal to know the experience and opinion of MDT coordinators on these matters so that we can understand what improves, or impairs the effectiveness of an MDT.

The aim of the survey reported here was to assess the views of MDT coordinators on their roles within the MDT. More specifically, the objective was to explore the views of the MDT coordinators on

1. The MDT decision making process and barriers to reach a decision.

2. Their role as coordinators and its requirements, with a focus on educational and training needs.

## Methods

### Study design

This was a prospective electronic survey study. The questions were structured for the purpose of the survey which was three-fold: first, to explore the prevalence of the role of the MDT coordinator in cancer MDTs nationally ( currently the UK Intercollegiate recommendations does not mandate that every MDT should have an MDT coordinator [7]). Secondly to explore their views, as core members, on the MDT efficacy and the process of decision making, and thirdly to explore their duties and requirements with focusing on training for the MDT coordinators. The development and implementation of training was given a priority by the ICC [7].

### Survey instrument

An online survey was administered electronically via freely available software ( http://www.surveymonkey.com). The survey comprised a total of 47 questions. Most of the questions were multiple choice questions; others required a yes or no answer. Respondents did not have to answer all the questions. Six demographic questions assessed the respondents’ background (professional group, gender, job title, age, work place and hospital type); Nine questions were about the MDT (e.g. venue, frequency and the MDT speciality(s) they coordinated); seven questions assessed the coordinators’ views on the discussion procedure of the MDT meeting (e.g. the frequency of the pathological or radiological images being displayed, if patients’ views, psychosocial or co-morbidities have been considered, and who contributes to decision making); five questions assessed respondents’ views on decision making processes and barriers thereof; eleven questions covered administering and coordinating of MDTs (e.g. the ease of preparing for the MDT, data recording and decision communication); five questions explored the coordinators views on MDT chairing and leadership (e.g. who chairs the MDT and whether the chair rotate or could/should rotate); and four questions assessed training for MDTs (e.g. have you had a training for your role? And in which areas do you think you need training?).

### Participants

A purposive sampling technique was used to target the population of interest. The National MDT Coordinators Forum is the national professional organisation for MDT coordinators in the UK. A link to the survey was sent to MDT coordinators, administrators and managers of all cancer types across the U.K via the National MDT Coordinators Forum. Survey recipients were asked to circulate the survey to other relevant MDT coordinators (snowballing sampling technique). The survey was completed between 6^th^ October and 6^th^ December 2010. Responses were anonymised, but a unique identifier was awarded to each respondent to enable comparison between respondents’ answers.

### Data analysis

All statistical analyses were performed using SPSS version 19.0 (SPSS Inc., Chicago, IL, USA).Descriptive statistics (means and standard deviations) were calculated for all survey items. Pearson Chi-Square was used to test whether there was an association between having undertaken training and stating that further training was required. Pearson Chi-square was also used to test for any association between role (MDT coordinators and their equivalents) and training received/further required. Statistical significance was taken at the 0.05 level.

The study was carried out in accordance with the declaration of Helsinki. This study was reviewed by the National Ethics Research Services NRES and deemed that ethics approval was unnecessary.

## Results

### Participants’ demographics

In total, there were 265 respondents to the survey. The majority of the responders were females (86%). The median age among the respondents was 40–49 years. 44.2%( n = 117) considered their hospitals to be a teaching hospitals, 9.8%( n = 26) from tertiary centres, 42.6%( n = 113) were from District General Hospitals (i.e., community hospitals across the UK). Table 1 shows the percentage of respondents who coordinate each tumour type (with some respondents coordinating more than one).

**Table 1:** Percentage of respondents who coordinate each tumour type (more than one response permitted)

**Area/Tumour type**	**%**	**Area/Tumour type**	**%**
Colorectal	17	Haematology	9
Upper Gastro-Intestinal	14	Skin	8
Gynaecology	14	Central Nervous System/Brain	4
Urology	13	Children	2
Lung	12	Endocrine	3
Breast	11	Palliative care	2
Head & Neck/Ear, Nose & Throat ENT	10		

### Job analysis

Regarding professional group, 82.6% (n = 219) reported themselves as MDT Coordinators (Group 1), the remaining (Group 2) were: 7.5% (n = 20) administrators, 3.4% (n = 9) cancer managers, 3.4% (n = 8) nurses and 3% (n = 8) were doctors (Grouping the respondents into Group 1 and 2 was to see if other health professionals’ views are similar to those who coordinate MDT meeting as their primary job). 52%( n = 138) of respondents had the title of MDT coordinator, whilst the remaining 48%( n = 127) had additional or alternative titles, which may explain why 37.7% (35.8% in Group 1 and 46.7% in group 2) felt that the job plan does not reflect their actual duties. Respondents had most commonly entered the position of MDT coordinator from other administrative positions in the NHS 41.5% (n = 110), but 11.7% (n = 31) had come from non-NHS jobs. 51.3% (n = 136) of the participants worked in satellite hospitals, 41.1%( n = 109) were from specialist cancer centres, and 8.7% (n = 23) worked in both. Regarding MDT work, 21.5% (n = 57) attended a MDT meeting more than once a week, 31.7% (n = 84) about once a week while 4.2% (n = 11) of the respondents attended once every 2 weeks.

### Respondents’ views on the MDT discussion

**Figure 1:**
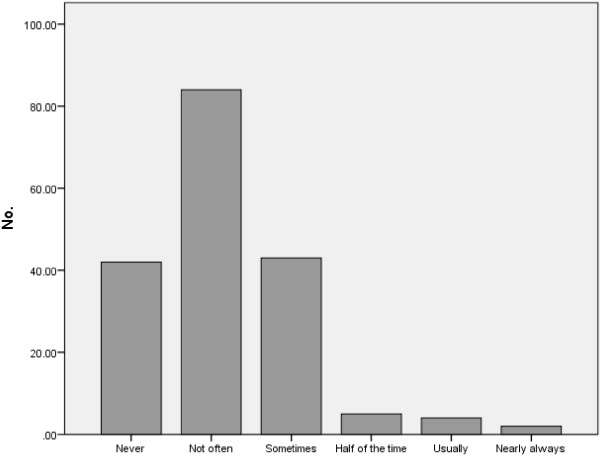
Respondents views on disagreement with MDT plan.

Respondents felt that medical members of the MDT (e.g., surgeons (41.5%), oncologists (32.8%)) always contribute to case discussions, whilst nurses nearly always (18%) contribute, and MDT Coordinators sometimes contribute (15.8%). Regarding the weight that the opinions of different MDT members have in deciding treatment decisions, surgeons’ opinions were deemed to always carry weight (23.4%), those of oncologists nearly always (13.2%), radiologists usually (11.3%), pathologists and nurses sometimes (12.1% and 17.7% respectively), and MDT coordinators never (37.7%). Nearly half of the Respondents felt that disagreements do not happen very often (Figure 1).

### MDT chairing and leadership*

For the position of chair of the MDT, 66.9% (n = 105) of respondents said the MDT meetings were chaired by surgeons, 33.8% (n = 53) physicians, 19.7% (n = 31) oncologists, 5.7% (n = 9) radiologists, 3.8% (n = 6) pathologists, 1.3% (n = 2) MDT coordinators and 0.6% (n = 1) cancer manager. 24% (n = 39) stated that the chair rotated between members, though 68% (n = 110) thought that the position of chair should rotate. When respondents were asked about how the meeting goes when the usual chair is away, 3% (n = 5) said better, 84% (n = 133) the same and 13% (n = 21) said worse.

*Some respondents selected more than one choice for MDT chairing hence the total number does not add up to 100%.

### Barriers to MDT decision making

**Figure 2:**
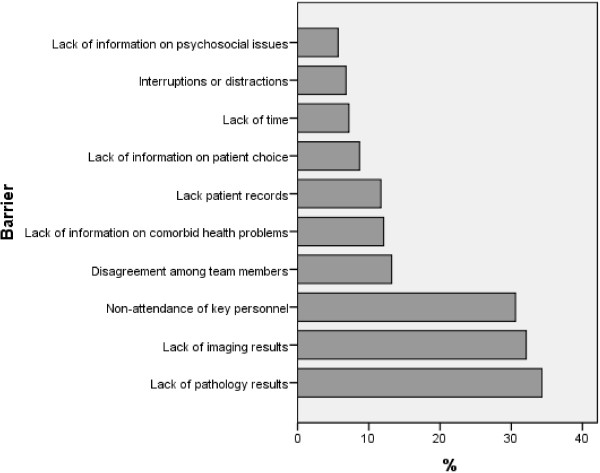
Barriers for reaching decision and MDT meeting.

The MDT coordinators reported that decisions for cancer patients are not formulated in the first presentation of all the cases at the MDT meeting. 46% of the respondents thought that nearly always a clear decision is made at the first presentation while 29% thought that there is usually a plan compared to 13% who said there is always a plan. There was no difference between the satellite hospital and the specialist centre in the frequency of reaching a clear plan in the first presentation of a case at the MDT. Respondents felt that when it was not possible to make decisions on cases at the first presentation, barriers were most commonly due to the lack of radiological or pathological information, or non-attendance of key personnel (Figure 2).

### Administering and coordinating cancer MDTs

**Figure 3:**
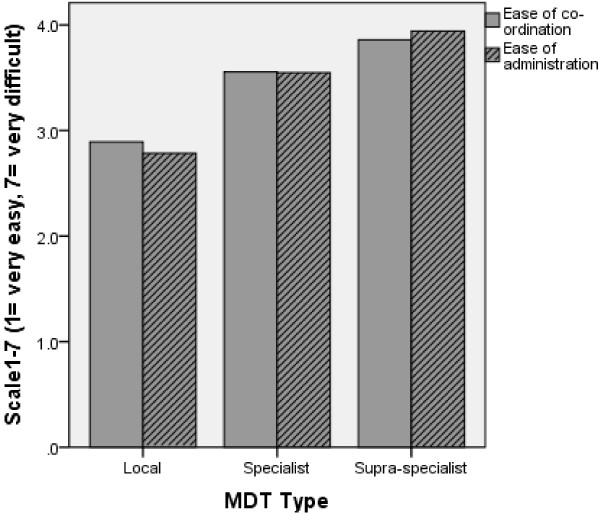
Ease of MDT meeting coordination and administration.

The survey results suggest that coordination and administration of the local MDT is easier than that of the specialist, or that of the supra-specialist MDTs (Figure 3). Different types of patient tracking software were reported available (patient tracking software is a computer systems that can be used to enter patient details including background history and investigation results. It also allows keeping patient records and MDT discussion and decision, in this case, efficiently i.e. a replacing the manual transcription and paperwork). Over 90% of respondents had patient tracking software at a local level, with this falling to 60% at specialist level and to less than 40% at supra specialist level.

Just under half of respondents were able to input data from MDT meetings in real time during the meetings. Fewer than 30% transpose data onto national databases. Regarding communication of the outcome of the MDT meeting, email was the most frequent means of communication to administrators and clinicians. Respondents tended to communicate to GPs by dictated letters. Approximately three quarters of respondents are able to send out decision or minutes from MDT meetings on the same day as the meeting, and over 90% within 48 hours, at a local level.

### Training

**Figure 4:**
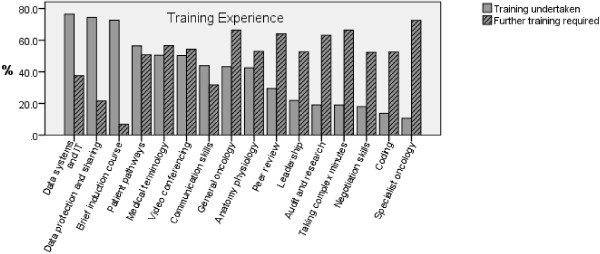
Training undertaken VS further training required for MDT coordinators.

The majority of respondents had undergone some kind of induction course and received training in Data systems and IT, data protection and sharing. The (Figure 4) outlines the areas in which training was undertaken and others that training needs are unmet and further training deemed required by the MDT coordinators.

There was no significant difference between group 1 (MDT coordinators) and group 2 (their equivalents) in receiving formal teaching in the areas of data system and IT (χ^2^ *= 0.917, P* = 0.33), General Oncology (χ^2 =^ 1.958, *P* = 0.16), Anatomy and physiology (χ^2 =^ 2.286, *P* = 0.13), Medical Terminology (χ^2 =^ 2.719, *P* = 0.09), videoconferencing ((χ^2 =^ 1.432, *P* = 0.231), brief induction course (χ^2 =^ 0.962, *P* = 0.327), patient pathways (χ^2 =^ 1.136, *P* = 0.287), taking complex minutes (χ^2 =^ 1.826, *P* = 0.177) and data protection (χ^2 =^1.032, *P* = 0.310). However, there was a significant association between group and training in receiving formal training in the following areas: specialist Oncology (χ^2 =^ 6.120, *P* = 0.013), coding (χ^2 =^ 10.064, *P* = 0.02), leadership (χ^2 =^ 27.0, *P* = <0.001), communication skills (χ^2 =^ 16.824, *P* < 0.001), audit and research (χ^2 =^ 25.18, *P* < 0.001), peer review (χ^2 =^ 12.673, *P* < 0.001) and negotiation skills (χ^2 =^ 19.097, *P* < 0.001). Interestingly, wherever there was a significant difference, group 1 had received less training than group 2. Finally, there was no significant difference between the 2 groups’ views in the requirement for further training which suggests that the role of MDT coordinator necessitates education and training regardless of the professional title of the person designated for this job.

## Discussion

Our survey of MDT coordinators, with the number and the geographical distribution of the responses, gives a nationally representative picture of MDT coordinators in UK. This is the second study of its kind, following a 2006 survey of the colorectal MDT coordinators [11].

Nearly half of the of the MDT coordinators felt that their job plan does not reflect what they actually do. MDT coordinators feel that they neither contribute to the MDT discussion nor their opinions carry weight in treatment decisions. Regarding leadership of MDT meetings, they thought that MDT meetings are mostly chaired by surgeons. MDT coordinators reported that there is not always a decision for each cancer case discussed at the MDT meeting and reasons for such are variable but lack of investigation results and non attendance of a key member were the commonest.

Respondents appear to have received some relevant training and have access to equipment and facilities appropriate for the job. A learning need analysis form a focus group study identified a need for an educational programme for MDT coordinators [12]. The UK Association of Cancer Registries (UKACR) produced a manual named the *MDT coordinator pack,* which is the available resource for training, besides what is locally arranged at each hospital. Recently a plan was put in place by the National Cancer Intelligence Network (NCIN) in collaboration with the UKACR, National Action Cancer Team (NCAT), Cancer Networks and the National Cancer MDT Coordinators Forum to produce an e-learning training programme aimed primarily at the MDT coordinators. This has led to the development of a tool *Understanding Cancer: Oncology Training for NHS and Public Health non-clinical staff* which was launched in April 2012 [13]. It can be said that sharing the result of this survey had a role in achieving the above. Although beyond the scope of this study, future research needs to investigate the training needs of MDT coordinators across different tumour types and to put into consideration the effects of case mix and inter compatibility of IT systems on training needs for organisational skills. There is evidence that the majority of domains of MDT working are common across major tumour types, but areas such as the clinical decision-making process have been found to vary and may need to be tailored to particular tumour types [14].

Comparing the results of our survey which was conducted in 2010 to the 2006 survey of the Colorectal cancer MDT coordinators [11] shows a marked improvement in the job related training received by the MDT coordinators and their equivalents for example compared to the 2006 survey, the percentage of MDT coordinators who had training has increased from 22% to 57.7% in Data system and IT, from 17.8% to 31.7% in general oncology and from 8.9% to 32.1% in anatomy/ physiology. We anticipate this educational resource will further enhance training for the MDT coordinators. An audit in a few years time would answer the question as to whether the training has improved for these MDT core members. Further, although MDT coordinators appear to not have a direct role in clinical decision-making, it seems that their work supports the decision-making of the clinical members of the MDT and without it decisions could not be made. These findings support our own previous research [15], as well as work carried out by others, including the National Cancer Action Team, and the ICCC of the Royal Colleges that recognises the role of the MDT coordinator, as well as the need to strengthen the position by improving resources and training available to MDT coordinators nationally [11,16,17]. This view has been reflected in the establishment of the National Team for the Cancer MDT coordinators (formally known as the Taskforce), Forum, and the Annual Conference, along with development of national job descriptions and training programmes [7]. Barriers to reach a clear decision at the MDT meetings are variable. Lack of Radiological and Pathological results at the time of the discussion followed by non attendance of MDT key members were the most common causes from the coordinators’ views in reaching a final decision. To a lesser extent they thought that unavailability or non-consideration of patients’ status and co-morbidities were other factors in the way of reaching such decisions. Similar results were observed in other studies where similar factors were blamed for not reaching a decision at the MDT meeting [10,18].

Certain limitations must be applied to our findings. There is no available statistical data on the total number of the MDT coordinators; however the majority of the 1500 MDTs in the UK are supported by a coordinator or an equivalent. Data on the educational background of coordinators was not gathered and may therefore be a confounder with regards to the level of training already undertaken as participants may have trained elsewhere prior to their work as MDT coordinators. The method used to recruit the survey sample involved snowballing so it is not possible to calculate the response rate. This means it is not possible to estimate the representativeness of responses. Furthermore, although the survey software records a unique identifier for each respondent, it is impossible to verify that each response is from a separate individual and therefore guarantee the integrity of the dataset. However, the sampling was successful in representing respondents across MDTs and different tumour types throughout the UK, and we have no reason to believe that respondents filed multiple surveys.

## Conclusions

This study raises the issue of training and education for the MDT coordinators. The study has shown that there is a normative, felt and an expressed need to train those key members of the MDT. Perhaps it is the time to seriously consider training or fill the gap in MDT coordinators’ education and training in the skills required for them to carry out their role with maximum effectiveness, with the view of further enhancing cancer care. We take the view that the MDT meeting should not be seen in isolation, but rather as a pivotal point in the patient care pathway, linking information about patients and their disease to the decision making process, and then to the ongoing care of the patient thereafter. The role of the MDT coordinator is therefore central to the care of cancer patients, both locally, and also through the coordination and sharing of data on a wider level.

## Abbreviations

MDT: Multidisciplinary team; ICCC: Intercollegiate cancer committee; SPSS: Statistical package for the social sciences; GP: General practitioner; IT: Information technology; UKACR: United kingdom association of cancer registries; NCIN: National cancer intelligence network; NCAT: National action cancer team.

## Competing interests

The authors declare that they have no competing interests.

## Authors’ contributions

The survey was conceived and designed by JG, BWL conducted the survey, RJ analysed the data and drafted the manuscript. SR helped with the statistical analysis and design of the manuscript, NS and JG helped draft the manuscript and revised the final manuscript. All authors read and approved the final manuscript.

## Pre-publication history

The pre-publication history for this paper can be accessed here:

http://www.biomedcentral.com/1472-6963/12/457/prepub
